# Vitamin D metabolites reshape bovine mammary epithelial responses relevant to mastitis through host-directed mechanisms

**DOI:** 10.3389/fcimb.2026.1815096

**Published:** 2026-05-01

**Authors:** Georgina Tiraboschi, Marilina Fernandez, Paula Isaac, María Laura Breser, Carina Porporatto, Luciana Paola Bohl

**Affiliations:** 1 Instituto Multidisciplinario de Investigación y Transferencia Agroalimentaria y Biotecnológica (IMITAB Consejo Nacional de Investigaciones Científicas y Técnicas - Universidad Nacional Villa María (CONICET-UNVM)), Villa María, Argentina; 2 Instituto Académico Pedagógico de Ciencias Básicas y Aplicadas, Universidad Nacional Villa María (UNVM), Villa María, Argentina; 3 Departamento de Biología Molecular, Facultad de Ciencias Exactas, Físico-Químicas y Naturales, Universidad Nacional de Río Cuarto (UNRC), Río Cuarto, Argentina; 4 Instituto de Biotecnología Ambiental y Salud (INBIAS-CONICET), Río Cuarto, Argentina

**Keywords:** bovine mastitis, host-directed therapy, immunomodulation, label-free quantitative proteomics, local immune response, MAC-T cells, *Staphylococcus aureus*, vitamin D_3_ compounds

## Abstract

**Introduction:**

Bovine mastitis remains one of the most prevalent diseases in dairy cattle and is largely managed through antibiotic therapy, which contributes to antimicrobial resistance. Host-directed strategies that enhance mammary epithelial defenses may complement conventional treatments. Although vitamin D_3_ metabolites can modulate immune responses, their comparative effects on bovine mammary epithelial cells have not been fully characterized. This study compared the antimicrobial and host-directed effects of vitamin D_3_ (D), 25-hydroxyvitamin D_3_ (25D), and 1α,25-dihydroxyvitamin D_3_ (1,25D) through an integrated *in vitro* approach.

**Methods:**

Antimicrobial and antibiofilm activity were assessed against mastitis-associated *Staphylococcus* spp. isolates. Bovine mammary epithelial MAC-T cells were then treated to evaluate cellular tolerance, transcriptional regulation of vitamin D-related enzymes and receptors, global proteomic alterations, and *Staphylococcus aureus* internalization.

**Results:**

D and 25D were well tolerated by MAC-T cells. They exhibited limited antimicrobial or antibiofilm activity, but pretreatment of the cells with 25D significantly reduced *S. aureus* internalization. Enzymes involved in vitamin D metabolism were differentially regulated by 25D and 1,25D: while 24-hydroxylase was upregulated, vitamin D receptor expression remained largely unchanged. Proteomic profiling identified 2,203 proteins common to all conditions, as well as compound-specific signatures related to epithelial homeostasis, innate immunity, vitamin D catabolism, vesicular trafficking, endocytic processes, and mitochondrial organization.

**Conclusion:**

The findings indicate that vitamin D_3_ metabolites primarily act through host-directed mechanisms rather than by direct antimicrobial activity. They could thus potentially be used as complementary strategies to manage mastitis in alignment with One Health principles.

## Graphical Abstract

**Figure:**
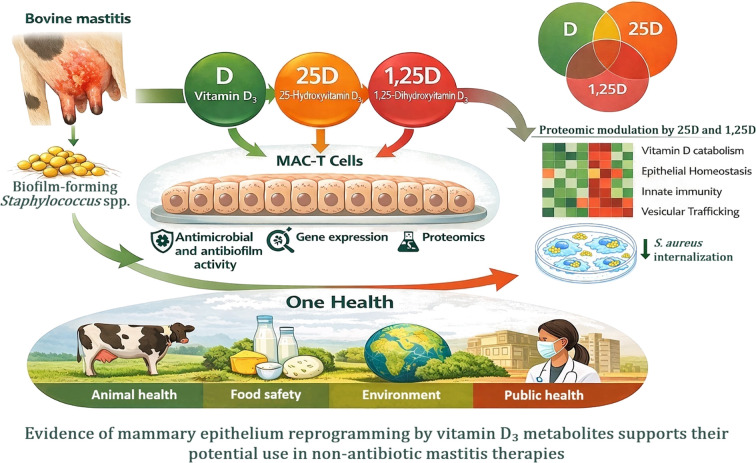

## 1 Introduction

Bovine mastitis costs the dairy industry tens of billions of dollars annually worldwide and represents a substantial economic burden for dairy farms (Morales-Ubaldo et al., 2023; Rasmussen et al., 2024; Rodriguez et al., 2024; Debruyn et al., 2025). It is primarily driven by *Staphylococcus* spp., which can evade antibiotics and establish themselves chronically by forming biofilm (Kerro Dego and Vidlund, 2024; Touaitia et al., 2025) and by invading the mammary epithelium. Increasing concerns over antimicrobial resistance have intensified the search for therapeutic agents capable of enhancing host defense mechanisms rather than exerting direct antibacterial activity.

Bovine mammary epithelial cells constitute the first barrier against invading pathogens and play an active role in innate immune responses within the mammary gland (Wellnitz and Bruckmaier, 2012). Beyond their structural function, they participate in pathogen recognition, cytokine production, the regulation of bacterial internalization, and the orchestration of local immune responses (Rainard et al., 2022; Huang et al., 2019). Consequently, mastitis management could benefit from strategies that modulate host-directed mechanisms in charge of epithelial cell function.

Although traditionally known for its role in calcium and phosphorus homeostasis, vitamin D (cholecalciferol) also regulates innate and adaptive immune responses through the activation of the vitamin D receptor (VDR), a ligand-dependent transcription factor expressed in bovine mammary epithelial cells. Its immunomodulatory properties have been demonstrated in different species, including cattle (Nelson et al., 2012; Hodnik et al., 2020; Eder and Grundmann, 2022; Tiraboschi et al., 2023). The intramammary infusion of one of its metabolites, 25-hydroxyvitamin D_3_ (calcidiol), significantly reduced milk bacterial counts and clinical symptoms in an acute *Streptococcus uberis* infection (Lippolis et al., 2011). The same researchers found that the treatment elicited host-defense gene expression (iNOS and β-defensins) and the modulation of neutrophil percentages (Merriman et al., 2017, 2018). Other studies have looked into the antimicrobial effects of vitamin D_3_ or its metabolites (calcidiol and 25D_2_) on the growth of *S. aureus* isolates from mastitis (Téllez-Pérez et al., 2012; Yue et al., 2017). After reporting a high prevalence of biofilm-forming *Staphylococcus* strains in local dairy farms (Felipe et al., 2017), our research group evaluated for the first time the direct and indirect antimicrobial and antibiofilm activity of another vitamin D metabolite, 1α,25-dihydroxyvitamin D_3_ or calcitriol (Tiraboschi et al., 2024).

However, the relative or comparative effects of these compounds remain relatively unexplored in bovine mastitis models. This is a relevant gap in knowledge, considering that they differ substantially in VDR affinity (1,25D_3_ > 25D_3_ > D_3_), as well as in their biological activity, plasma concentrations, commercial costs, and toxicity risk. The latter is especially true in the case of systemic 1,25D_3_, which can lead to hypercalcemia (Tiraboschi et al., 2023; Bikle, 2025). A study seeking to undertake such a comparison would benefit from incorporating proteomics into its approach, since it can offer unique insights into vitamin D-mediated responses beyond transcriptional regulation. Indeed, proteomics could make it possible to understand whether and how various vitamin D compounds differentially alter protein abundance and drive post-translational modifications in the bovine mammary epithelium in the absence of direct infection. The characterization of these responses, which is currently lacking, would go a long way towards understanding the different modes of action of vitamin D compounds and how they could best be used to treat bovine mastitis.

Against this background, the present study aimed to: (1) evaluate the activity of vitamin D_3_ and 25-hydroxyvitamin D_3_ against *Staphylococcus* isolates from bovine mastitis, so as to complement our earlier results for calcitriol; (2) characterize the transcriptional regulation of vitamin D-related genes and the overall proteomic changes in response to treatment with vitamin D_3_, 25-hydroxyvitamin D_3_, or 1α,25-dihydroxyvitamin D_3_ in MAC-T cells, and (3) assess the effect of vitamin D_3_ and 25-hydroxyvitamin D_3_ on the internalization of *S. aureus* into pre-treated MAC-T cells. In short, the study sought to compare the three compounds in terms of their antimicrobial and antibiofilm activity, their ability to prevent *S. aureus* internalization, and their impact on the epithelial proteome and host-mediated proteomic mechanisms.

## 2 Materials and methods

### 2.1 Reagents, bacterial strains, cell lines and culture conditions

Vitamin D_3_, 25-hydroxyvitamin D_3_, and 1,25-dihydroxyvitamin D_3_ were purchased from Cayman Chemical (Ann Arbor, MI, USA). From here on, these compounds will be referred to simply as D, 25D and 1,25D. In earlier research (Tiraboschi et al., 2024), our group studied the antimicrobial and antibiofilm activity of 1,25D against *Staphylococcus* isolates from bovine mastitis, as well as cellular tolerance and *S. aureus* internalization in MAC-T cells treated with 1,25D. The same methodology was applied here to assess D and 25D, whereas all three compounds were subjected to the transcriptional and proteomic analyses described later on. Stock and working solutions were prepared in isopropyl alcohol (Anedra by Research AG, Buenos Aires, Argentina), which was the vehicle in all the controls, at final concentrations not exceeding 0.5% (v/v). These concentrations were chosen on the basis of our own results (Tiraboschi et al., 2024) and other relevant literature (Téllez-Pérez et al., 2012; Alva-Murillo et al., 2014; Yue et al., 2017). Even though they all remained above physiological plasma levels (1,25D: ~0.05-0.12 nM; 25D: 20~90 nM; D: ~4 nM, Tiraboschi et al., 2023), substantially supraphysiological concentrations posing a risk of hypercalcemia were avoided. For a full list of the concentrations used in each assay, see **Table 1**.

**Table 1:** Concentrations of vitamin D compounds used in each experimental assay.

Assay Compound	Antimicrobial activity	Antibiofilm activity	Cytotoxicity	Quantitative PCR analysis	Proteomics	Internalization
D (nM)	10-200	25-200	25-200	50	50	25-100
25D (nM)	100-2000	250-2000	250-2000	500	500	125-500
1,25D (nM)	-	–	–	100	100	–

1,25D was previously tested against *Staphylococcus* spp. from bovine mastitis (antimicrobial/antibiofilm assays) and in MAC-T cells (tolerance/internalization) by our group (Tiraboschi et al., 2024).

On the other hand, four biofilm-forming *Staphylococcus* spp. previously isolated from cattle with mastitis were used: *S. aureus* V329 (Cucarella et al., 2001) and the non-aureus isolates *S. chromogenes* 40, *S. xylosus* 4913, and *S. haemolyticus* 6 (Felipe et al., 2017). Bacterial cultures were maintained in trypticase soy broth or agar (TSB/TSA; Britania, Buenos Aires, Argentina) under routine laboratory conditions.

The chosen bovine mammary epithelial cell line was MAC-T (Huynh et al., 1991). Culture conditions replicated those described in an earlier study by our group (Isaac et al., 2017). The cells were maintained in Dulbecco’s modified Eagle medium (DMEM; Life Technologies, NY, USA) supplemented with 10% (v/v) fetal bovine serum (Natacor, Córdoba, Argentina) and CTS™ GlutaMAX™-I (1:100; Life Technologies, Grand Island, NY, USA).

### 2.2 Antimicrobial and antibiofilm activity by D and 25D

The influence of D and 25D on the growth kinetics of the staphylococcal isolates was evaluated as reported earlier (Téllez-Pérez et al., 2012). Briefly, bacterial suspensions adjusted to 0.5 McFarland in TSB were distributed into 96-well polystyrene microplates containing D (10, 25, 50, 100, or 200 nM) or 25D (100, 250, 500, 1000, or 2000 nM). The plates were incubated at 37 °C for 12 h. Cloxacillin (8 µg/mL; Sigma-Aldrich, St. Louis, MO, USA) was included as a growth inhibition control. Growth curves were built on the basis of optical density measurements, recorded at 620 nm at 1 h intervals with a Multiskan™ FC microplate reader (Thermo Fisher Scientific, Shanghai, China). Specific growth rates were subsequently calculated from the slope of the linear region corresponding to the exponential growth phase, as previously described (Matos et al., 2008).

The effects of D and 25D on biofilm formation and established biofilms were also assessed. For the biofilm development assay, bacterial suspensions standardized to 0.5 McFarland were dispensed (100 µL per well) into 96-well polystyrene microplates containing D (final concentrations of 25, 50, 100, or 200 nM) or 25D (final concentrations of 250, 500, 1000, or 2000 nM). The plates were incubated for 24 h. For the mature biofilm assay, bacterial inocula (200 µL; 0.5 McFarland) were first incubated in 96-well plates for 24 h to allow biofilm to establish itself. Non-adherent cells were subsequently removed by washing twice with phosphate-buffered saline (PBS). After that, D or 25D were added and incubated with the preformed biofilms for an additional 24 h.

Biofilm biomass was quantified through crystal violet staining, as in O'Toole and Kolter (1998). Following treatment, the wells were gently washed three times with sterile PBS, and the remaining adherent cells were heat-fixed at 60 °C for 1 h until complete drying. The biofilms were then stained with 0.1% (w/v) crystal violet solution (Anedra) for 15 min. Excess dye was removed with distilled water and the bound stain was solubilized with 97% ethanol (Anedra) for 30 min. Aliquots (100 µL) were transferred to fresh microplates, and absorbance was measured at 570 nm using a Multiskan™ FC microplate reader. Data were expressed as the percentage of absorbance relative to the vehicle-treated control, which was considered to represent 100% absorbance.

### 2.3 Tolerance of bovine mammary epithelial cells to D and 25D

The cytotoxic effects of D and 25D were assessed in the MAC-T cell line. Cells were seeded at a density of 5 × 10^4^ cells per well in 96-well plates and incubated for 24 h at 37 °C. Cells were exposed to D (25, 50, 100, or 200 nM) or 25D (250, 500, 1000, or 2000 nM) for 24 h or 72 h. Cell viability was determined with a thiazolyl blue tetrazolium bromide (MTT) assay, as in Isaac et al. (2017). After treatment, the culture media were removed and replaced with MTT solution (0.5 mg/mL; Sigma-Aldrich), and the cells were incubated for 4 h at 37 °C in the dark. The MTT solution was then discarded, and the resulting formazan crystals were dissolved in dimethyl sulfoxide (DMSO; Biopack, Buenos Aires, Argentina). Absorbance was measured at 570 nm with a Multiskan™ FC microplate reader. Triton X-100 (1% v/v; Sigma-Aldrich) was used as a positive control for cytotoxicity. Cell viability was expressed as a percentage relative to the vehicle-treated control, which was considered to represent 100% viability.

### 2.4 Expression of vitamin D-related enzymes and receptor

The expression of vitamin D-related hydroxylases and of the VDR was evaluated by reverse transcription quantitative polymerase chain reaction (RT-qPCR), following Bohl et al. (2021). Briefly, MAC-T cells were treated for 12 h with D (50 nM), 25D (500 nM), or 1,25D (100 nM). Isopropanol was the vehicle in the control. The relative mRNA levels of the genes encoding 25-hydroxylase, 1α-hydroxylase, 24-hydroxylase, and VDR were determined.

Total RNA was isolated from the cells using the EasyPure RNA kit (TransGen Biotech Co., LTD., Beijing, China), which includes a DNase treatment, according to the manufacturer’s instructions. RNA quantity and quality were assessed with a microliter spectrophotometer (Picodrop, Hinxton, UK). RT-qPCR reactions were carried out using 100 ng of total RNA with the iTaq Universal SYBR^®^ Green One-Step Kit (Bio-Rad Laboratories, Hercules, CA, USA) in a CFX96 Touch™ Real-Time PCR Detection System (Bio-Rad Laboratories). Reactions were performed according to the manufacturer’s instructions with minor modifications, using an annealing temperature of 60 °C. No-template controls (NTC) were included in each RT-qPCR run to monitor potential contamination, and all the reactions were run in duplicate. Amplicon specificity was verified by melting-curve analysis.

Glyceraldehyde-3-phosphate dehydrogenase (GAPDH) was the reference gene. **Supplementary Table 1** contains the primer sequences, amplicon sizes, and literature referencing all the target genes. Relative gene expression was calculated using the 2^−^ΔΔCt method (Livak and Schmittgen, 2001) and expressed as fold changes relative to the control group.

### 2.5 Comparative quantitative proteomic analysis of bovine mammary epithelial cell lysates after exposure to vitamin D compounds

#### 2.5.1 Preparation of MAC-T cell lysates for proteomic analysis and sodium dodecyl sulfate-polyacrylamide gel electrophoresis

MAC-T cells (1.2 × 10^6^ cells) were seeded in 100-mm culture dishes in complete medium, as described above, and grown to confluence. Next, the cells were washed with PBS and treated for 24 h with D (50 nM), 25D (500 nM), or 1,25D (100 nM). Isopropanol (0.1%) was the vehicle in the control. The treatments were prepared in culture medium without fetal bovine serum to prevent interference with the downstream proteomic analysis.

After treatment, the cells were washed three times with PBS and lysed with 800 µL of lysis buffer per plate (PBS containing 1% Triton X-100 and Protease Inhibitor Cocktail P2714, 1×; Sigma-Aldrich). The plates were incubated for 10 min at 4 °C, after which the cells were detached with a cell scraper. The lysates were transferred to microcentrifuge tubes and centrifuged at 10,000 rpm for 15 min at 4 °C. The supernatants were recovered and total protein concentration was determined through a bicinchoninic acid (BCA) microplate assay (Thermo Scientific™ Pierce™ BCA Protein Assay Kit, Cat. No. 23225), following the manufacturer’s instructions. Samples and bovine serum albumin standards were incubated with the working reagent at 37 °C for 30 min, and absorbance was measured at 562 nm using a Multiskan™ FC microplate reader. Protein concentrations were calculated from a standard curve after blank subtraction.

Protein separation was carried out by sodium dodecyl sulfate–polyacrylamide gel electrophoresis (SDS–PAGE) following Maizel (2000), with some modifications by Ballatore et al. (2020). All the steps were performed as indicated in the guidelines of the CEQUIBIEM proteomics facility (http://cequibiem.qb.fcen.uba.ar), where the analyses were conducted. Briefly, 50 µg of total protein from treated MAC-T cell lysates were mixed with 5× sample buffer, heat-denatured at 80 °C for 10 min, and separated on 5% stacking and 15% resolving gels using a Mini-PROTEAN Tetra System (Bio-Rad Laboratories). Electrophoresis was run at constant current until the proteins had migrated approximately 1 cm into the resolving gel. The gels were then fixed, stained with Coomassie Brilliant Blue G-250 (Anedra), and washed. Finally, protein bands were excised for subsequent mass spectrometry (MS).

#### 2.5.2 Mass spectrometry analysis and data processing

MS was performed at CEQUIBIEM, in an EASY-nLC 1000 system coupled to a Q-Exactive Orbitrap mass spectrometer (Thermo Fisher Scientific, Waltham, MA, USA). The excised Coomassie-stained SDS-PAGE gel protein bands were sequentially washed and de-stained with 50 mM ammonium bicarbonate, followed by 25 mM ammonium bicarbonate in 50% acetonitrile, and finally 100% acetonitrile. The proteins were reduced with 10 mM of dithiothreitol at 56 °C for 45 min, followed by alkylation with 20 mM of iodoacetamide at room temperature for 45 min in the dark. Afterwards, they were digested with trypsin (Promega V5111, Madison, WI, USA) at a 1:50 enzyme-to-protein ratio in 50 mM of ammonium bicarbonate (pH 8.0), and incubated overnight at 37 °C. The peptides were desalted with C18 resin (Merck, Darmstadt, Germany), eluted with 50% acetonitrile (ACN):0.5% trifluoroacetic acid, dried in a SpeedVac concentrator, and stored at -20 °C. Prior to MS, the dried peptides were reconstituted in 30 µL of 0.1% formic acid (FA).

Tryptic peptides were loaded onto an EASY-Spray Accucore C18 analytical column (25 cm × 75 μm i.d., 2 μm particle size, 100 Å pore size; P/N ES902; Thermo Fisher Scientific). They were eluted through the nanoLC system using the following gradient at a flow rate of 300 nL/min: 7% buffer B (ACN and 0.1% FA) between 0–5 min, 35% buffer B between 5–105 min, 95% buffer B between 105–110 min, and 95% for the last 10 min. The corresponding data were subsequently acquired using a data-dependent acquisition (DDA) mode. For that, the electrospray voltage was set to 3.5 kV, with a gas flow rate of 3.0 L/min at 180 °C. Full MS data were acquired in a mass range of 400–2000 *m*/*z*.

The raw DDA-MS files were compared on Proteome Discoverer v2.2 (Thermo Fisher Scientific) against an *in silico-*predicted spectral library based on the *Bos taurus* (Bovine) UniProt database (UP000009136; 161,005 entries; release 24-04-2024). Protease was set to ‘Trypsin’ with one missed cleavage allowed. Other parameters for the analysis included a precursor *m*/*z* range of 400–2000, fragment *m*/*z* range of 200-1800, precursor charge states of 2-4, and peptide lengths between 7 and 30 amino acids. Fixed modifications included the carbamidomethylation of cysteine, whereas the oxidation of methionine was set as a variable modification. Mass accuracy was set to 10 ppm for precursor ions and to 0.05 Da for fragment ions. A minimum of two peptides per protein was required for identification. Protein hits were filtered for high confidence peptide matches, with a maximum protein and peptide false discovery rate of 1% calculated through a reverse database strategy.

Proteome Discoverer calculated an average area for each protein under each condition, on the basis of the area under the curve of the three most intense unique peptides per protein. These calculations were made for three technical replicates per condition and normalized.

The data were post-processed and statistically analyzed on Perseus v1.6.6.0 Max Planck Institute of Biochemistry (Tyanova et al., 2016). Reproducibility among biological replicates was assessed for each condition (**Supplementary Table 2**). The following steps were performed for data filtering: (i) logarithmic transformation of normalized abundances (log_2_), (ii) acceptance of valid values in at least two out of three biological replicates, and (iii) imputation of missing peptide intensities through replacement with values drawn from a normal distribution modeled on the dataset. This last step was only carried out for proteins detected under all conditions.

Treatments were compared pairwise using Student’s t-test, and volcano plots were generated by plotting −log_10_(p-value) against log_2_ fold change. Proteins with a log_2_ fold change ≥ 1 (equivalent to a fold change ≥ 2) were classified as upregulated, while those with a log_2_ fold change ≤ –1 were considered to be downregulated. Statistical significance was determined using an adjusted p-value ≤ 0.05 (corresponding to –log_10_(p) ≥ 1.3 on the y-axis).

Proteins exhibiting significant variation across the four conditions were identified with a one-way analysis of variance test (ANOVA). Only proteins with p-values ≤ 0.05 were considered significantly regulated and used to create a heatmap on RStudio (RStudio Team, 2024). This heatmap made it possible to visualize regulation patterns across conditions based on log_2_ fold changes, as described above.

The biological interpretation of the results was aided by g:Profiler (Kolberg et al., 2023) and UniprotKB (Ahmad et al., 2025), on the basis of Gene Ontology (GO) annotations and biological pathway information. According to GO, proteins were categorized into three functional groups: biological process (BP), cellular component (CC), and molecular function (MF). Enrichment of GO terms via g:Profiler was assessed with the hypergeometric test, and statistical significance was determined using a Benjamini-Hochberg (BH) adjusted *p*-value threshold of 0.05. In addition, protein-protein interaction networks were explored using the STRING database to evaluate functional connectivity among differentially regulated proteins (Szklarczyk et al., 2023).

The MS proteomic data were deposited to the ProteomeXchange Consortium via the partner repository PRIDE, under the dataset identifier [PXD071673].

### 2.6 Bacterial internalization assay

Bacterial internalization into MAC-T cells was evaluated through a gentamicin protection assay, following Bohl et al. (2021). The cells were pretreated for 24 h with D (25, 50, or 100 nM) or 25D (125, 250, or 500 nM) and subsequently infected with *S. aureus* V329 at a multiplicity of infection of 30. After 2 h of incubation, extracellular bacteria were eliminated by treatment with gentamicin (100 μg/mL) for 1 h and intracellular bacteria were quantified following host cell lysis. The effectiveness of the gentamicin treatment was verified by plating supernatant aliquots on TSA plates and incubating them at 37 °C for 24 h. Internalization levels were expressed as percentages relative to vehicle-treated control cells, which were considered to represent 100% internalization.

### 2.7 Statistical analysis

With the exception of the proteomics assays (in which three technical replicates were made from one biological preparation, statistics in M&M), all the experiments were independently performed on three separate occasions, with each experiment conducted in triplicate. Prior to statistical evaluation, some variables were transformed and expressed as percentages relative to the corresponding control. Differences between groups were assessed by ANOVA followed by Bonferroni’s *post hoc* test, and values of p < 0.05 were considered statistically significant. The data were statistically analyzed on Infostat v2020 (Di Rienzo et al., 2020) and are presented as mean values with their corresponding standard errors (SE).

## 3 Results

### 3.1 D and 25D did not exhibit direct antimicrobial or antibiofilm activity

**Figure 1:**
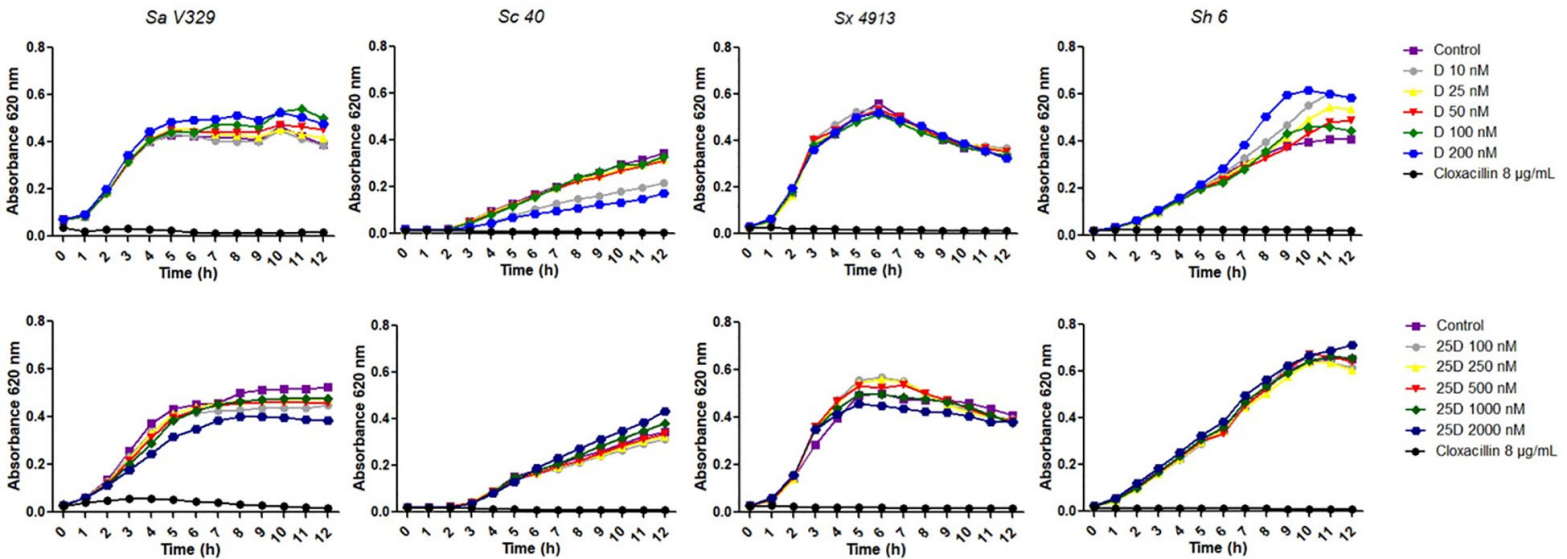
Growth response of *Staphylococcus* spp. to vitamin D_3_ and 25-hydroxyvitamin D_3_. Cultures of *S. aureus* V329 (Sa V329), *S. chromogenes* 40 (Sc 40), *S. xylosus* 4913 (Sx 4913) and *S. haemolyticus* 6 (Sh 6) were incubated in TSB supplemented with increasing concentrations of vitamin D_3_ (D) or 25-hydroxyvitamin D_3_ (25D). Cloxacillin (8 µg/mL) was included as a positive antimicrobial control. The plots display mean absorbance values at 620 nm.

Given that vitamin D and its metabolites have been proposed to influence host-pathogen interactions in bovine mastitis, we first evaluated whether D and 25D had direct antimicrobial or antibiofilm effects against mastitis-associated *Staphylococcus* isolates. The growth rates of isolates exposed to D remained similar to those of the untreated controls at all the concentrations tested, i.e. D did not modify growth kinetics in any case (**Figure 1**; **Table 2**). In contrast, specific concentrations of 25D caused a modest but detectable reduction in the growth rate of *S. aureus* V329, *S. chromogenes* 40, and *S. xylosus* 4913 (**Figure 1**; **Table 2**). Although these changes were statistically significant with respect to the control, they were much less marked than the inhibitory effect produced by cloxacillin. The findings indicate that neither D nor 25D had genuine antimicrobial activity under the conditions tested, and that the slight growth delays induced by 25D did not translate into biologically meaningful inhibition.

**Table 2:** Growth rates of *Staphylococcus* isolates.

Treatment	*Sa V329*	*Sc 40*	*Sx 4913*	*Sh 6*
Control	0.108 ± 0.001 B	0.036 ± 0.001 B	0.154 ± 0.009 BC	0.050 ± 0.003 B
D 10 nM	0.108 ± 0.001 B	0.025 ± 0.005 B	0.176 ± 0.005 C	0.051 ± 0.004 B
D 25 nM	0.111 ± 0.001 B	0.035 ± 0.001 B	0.166 ± 0.004 BC	0.047 ± 0.004 B
D 50 nM	0.109 ± 0.003 B	0.036 ± 3,3e-04 B	0.173 ± 0.002 BC	0.044 ± 0.006 B
D 100 nM	0.110 ± 0.002 B	0.038 ± 0.002 B	0.160 ± 0.002 BC	0.042 ± 0.003 B
D 200 nM	0.120 ± 0.001 C	0.016 ± 0.011 AB	0.149 ± 0.006 B	0.054 ± 0.006 B
cloxacillin 8 μg/mL	0.002 ± 0.000 A	-0.001 ± 1,2e-04 A	-0.003 ± 0.000 A	2,7e-04 ± 3,3e-05 A
Control	0.098 ± 0.002 D	0.039 ± 0.001 CD	0.114 ± 0.002 C	0.055 ± 0.002 B
25D 100 nM	0.088 ± 0.002 CD	0.033 ± 3,3e-04 B	0.134 ± 0.001 D	0.067 ± 0.004 BC
25D 250 nM	0.091 ± 0.002 CD	0.034 ± 0.001 B	0.130 ± 0.002 D	0.067 ± 0.004 BC
25D 500 nM	0.086 ± 0.004 CD	0.035 ± 0.001 BC	0.128 ± 0.003 D	0.067 ± 0.003 BC
25D 1000 nM	0.082 ± 0.004 C	0.041 ± 0.000 D	0.116 ± 0.001 C	0.071 ± 0.003 C
25D 2000 nM	0.064 ± 0.003 B	0.047 ± 0.001 E	0.104 ± 0.000 B	0.072 ± 0.001 C
cloxacillin 8 μg/mL	0.002 ± 0.001 A	-0.002 ± 3,3e-04 A	-0.002 ± 0.000 A	-0.001 ± 0.000 A

Suspensions of *S. aureus* V329 (Sa V329), *S. chromogenes* 40 (Sc 40), *S. xylosus* 4913 (Sx 4913), and *S. haemolyticus* 6 (Sh 6) were exposed to vitamin D_3_ or 25-hydroxyvitamin D_3_. Growth rate values are presented as means ± SE. Data were analyzed by ANOVA followed by Bonferroni’s *post hoc* test. Statistical significance was set at p < 0.05. Different letters indicate statistically significant differences between treatments within each bacterial species for a given vitamin D compound.

D, vitamin D3; 25D, 25-hydroxyvitamin D3.

**Figure 2:**
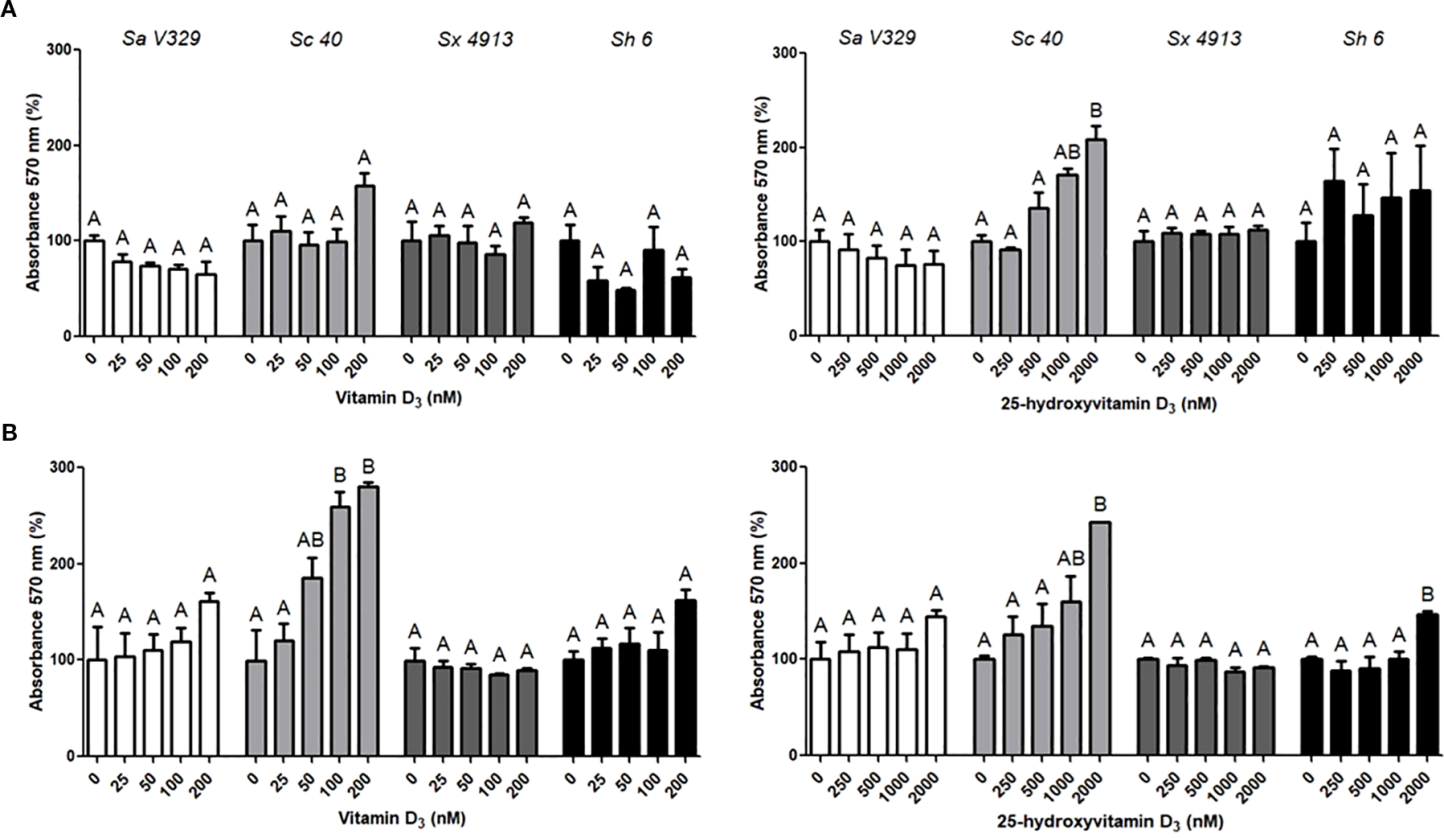
Effects of vitamin D_3_ and 25-hydroxyvitamin D_3_ on biofilm formation **(A)** and on preformed biofilms **(B)**. **(A)** *S. aureus* V329 (Sa V329), *S. chromogenes* 40 (Sc 40), *S. xylosus* 4913 (Sx 4913), and *S. haemolyticus* 6 (Sh 6) were incubated with vitamin D_3_ (0–200 nM) or 25-hydroxyvitamin D_3_ (0–2000 nM) in TSB for 24 h, and biofilm biomass was quantified by crystal violet staining. **(B)** Pre-established biofilms of the same isolates were exposed to the same concentration ranges of each compound for 24 h. After that, residual biofilm biomass was assessed by crystal violet staining. In both panels, data represent the percentage of absorbance at 570 nm relative to the vehicle-treated control (100% absorbance). Values correspond to means ± SE. Data were analyzed by ANOVA followed by Bonferroni’s *post hoc* test. Differences were considered significant at p < 0.05, and different letters indicate statistically significant differences between treatments within each bacterial species for a given compound.

In agreement with the lack of antimicrobial activity, none of the concentrations of D or 25D reduced biofilm formation or the biomass of preformed biofilms for any of the *Staphylococcus* isolates tested (**Figure 2**). Similarly, growth-curve data showed that D had no impact on bacterial proliferation, while 25D produced only slight, strain-specific delays that did not translate into measurable antibiofilm effects under the conditions evaluated.

### 3.2 D and 25D were well tolerated by bovine mammary epithelial cells

**Figure 3:**
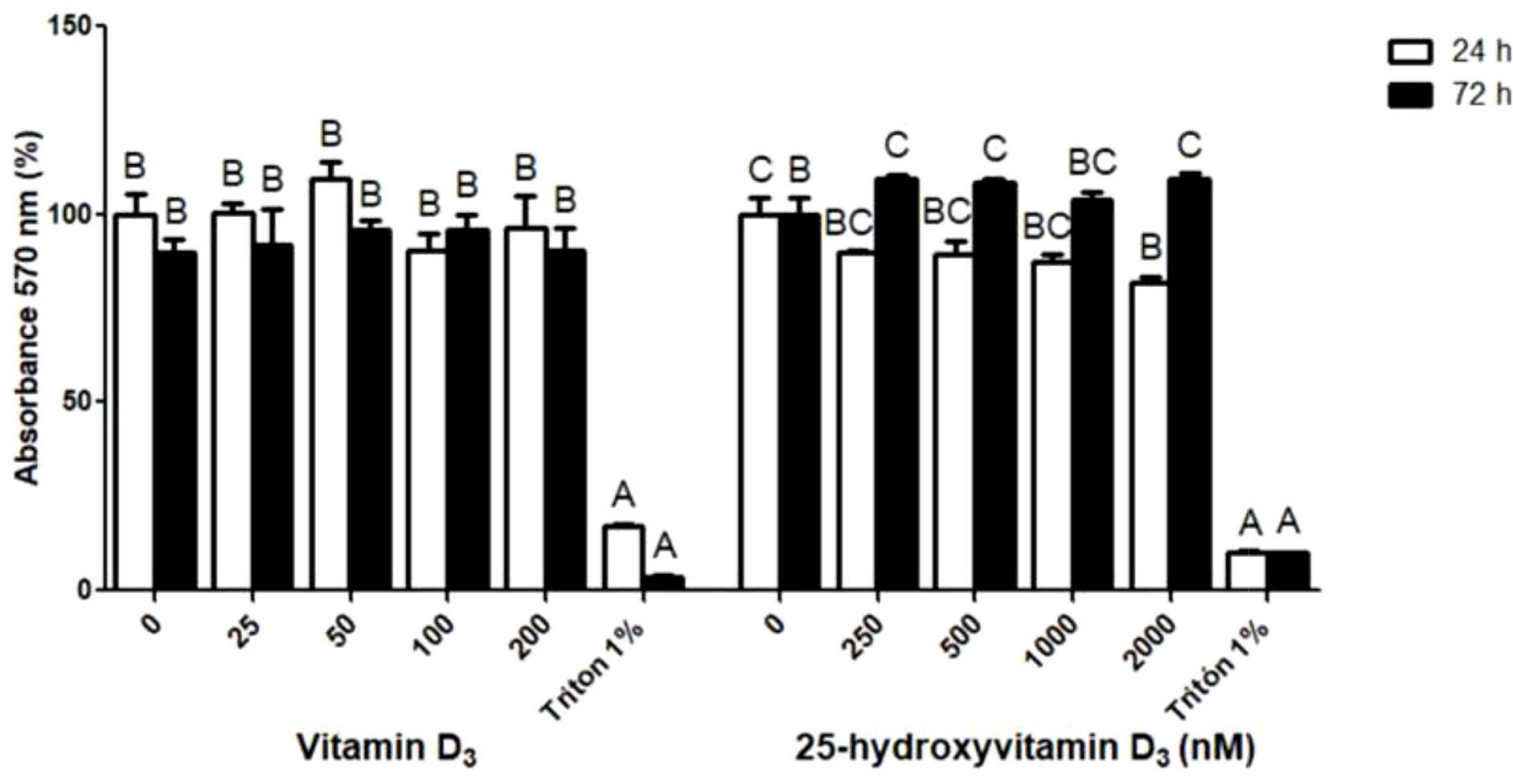
Viability of bovine MAC-T cells following exposure to vitamin D_3_ and 25-hydroxyvitamin D_3_. Cells were treated for 24 h or 72 h and viability was assessed using a MTT assay. Triton X-100 (1% v/v) served as the cytotoxicity control. Results are presented as percentages relative to vehicle-treated cells, whose viability was defined as 100%. Data represent the mean ± SE. Statistical analysis was performed by ANOVA followed by Bonferroni’s *post hoc* test, considering p < 0.05 as significant. Distinct letters denote statistically significant differences between treatments within each metabolite and point in time.

The viability of MAC-T cells was not affected by D at any of the concentrations or points in time evaluated with respect to the control (**Figure 3**). A slight but detectable decrease in cell viability was registered after 24 h of treatment with 2000 nM of 25D, but control-like values returned after 72 h. No other concentrations of 25D altered cell viability (**Figure 3**). In short, both compounds were well tolerated by bovine mammary epithelial cells under the conditions tested, i.e. they are potentially safe for administration in bovine systems.

### 3.3 Vitamin D-related enzymes and receptor expression were differentially modulated by D, 25D and 1,25D in MAC-T cells

Treatment with 25D resulted in a statistically significant upregulation of both 25-hydroxylase and 24-hydroxylase mRNA levels with respect to cells treated with D and the control (**Table 3**). The magnitude of this upregulation, as reflected by the fold-change values, was greater for 24-hydroxylase than for 25-hydroxylase. Similarly, exposure to 1,25D led to a significant increase in 24-hydroxylase expression with respect to both control and D-treated cells. In contrast, no statistically significant differences were observed in 1α-hydroxylase or VDR expression after any of the treatments in comparison with the control (**Table 3**).

**Table 3:** RT-qPCR analysis of vitamin D-related genes in MAC-T cells following 12 h of treatment with vitamin D compounds.

Treatment	25-hydroxylase	1α-hydroxylase	24-hydroxylase	VDR
Control	1 ± 0.1 A	1 ± 0.09 A	1 ± 0.05 A	1 ± 0.1 A
D	1.09 ± 0.07 A	0.88 ± 0.1 A	0.5 ± 0.1 A	1.19 ± 0.51 A
25 D	2.98 ± 0.47 B	1.45 ± 0.42 A	102.68 ± 20.46 B	0.45 ± 0.11 A
1,25 D	2.08 ± 0.56A B	1.23 ± 0.04 A	79.61 ± 13.55 B	0.46 ± 0.15 A

Relative mRNA expression of 25-hydroxylase, 1α-hydroxylase, 24-hydroxylase, and VDR was quantified using the 2^−^ΔΔCt method. GAPDH served as the endogenous reference gene. Data are expressed as mean fold change ± SE relative to vehicle-treated controls. ANOVA was performed followed by Bonferroni’s *post hoc* test, considering p < 0.05 as significant. Different letters indicate significant differences between treatments for each gene. D, vitamin D_3_ 50 nM; 25D, 25-hydroxyvitamin D_3_ 500 nM; 1,25D, 1α,25-dihydroxyvitamin D_3_ 100 nM.

Overall, the RT-qPCR analysis demonstrated that the two hydroxylated metabolites elicited a selective transcriptional response in the cells, characterized primarily by the induction of genes involved in vitamin D metabolism, particularly 24-hydroxylase. Based on these transcriptional findings, subsequent proteomic analyses were conducted to assess whether these regulatory effects were also observed at the protein level, as well as to explore broader cellular pathways modulated by the vitamin D compounds.

### 3.4 Treatment with vitamin D compounds is associated with differential proteomic changes in MAC-T cells

**Figure 4:**
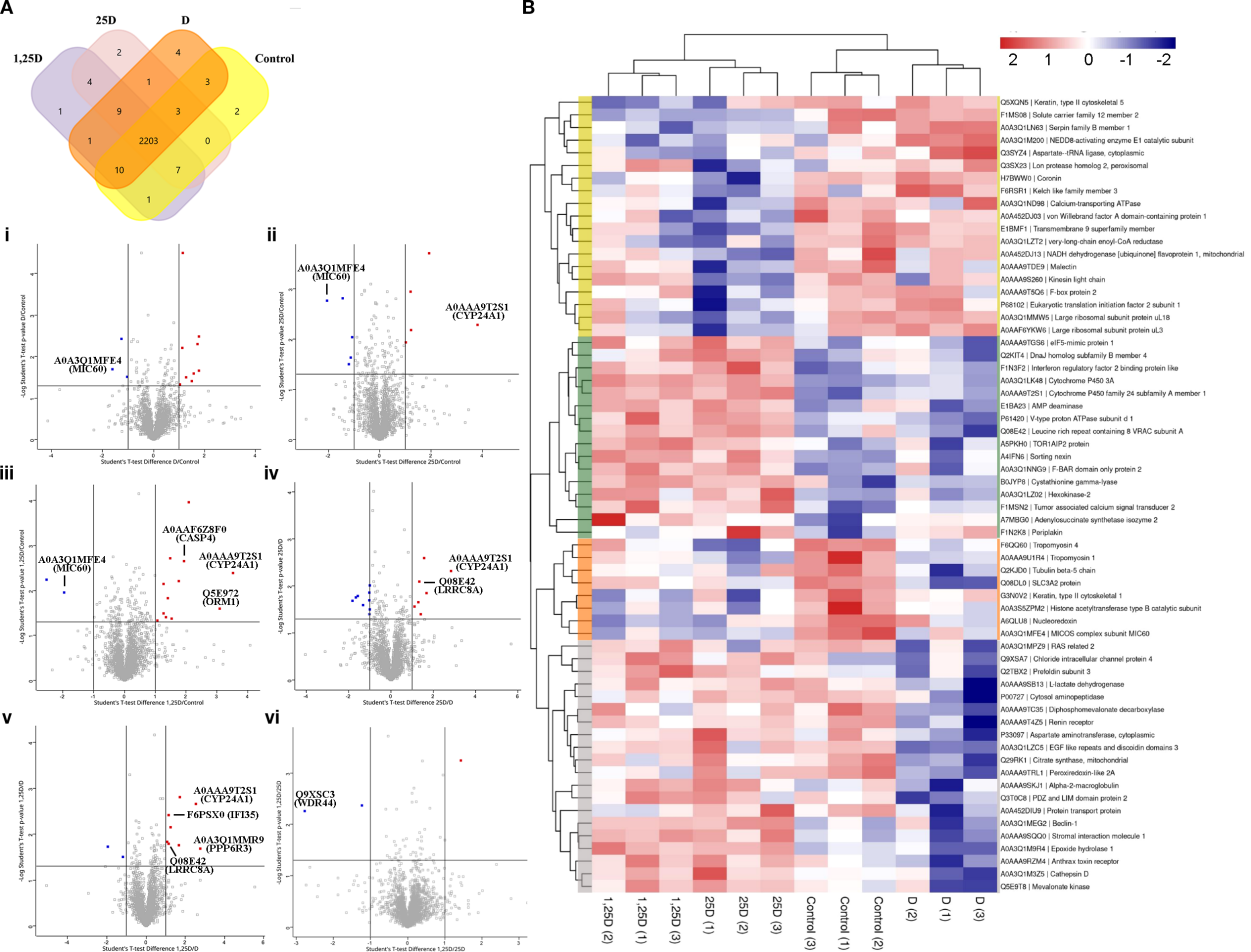
Proteomic profiling of bovine mammary epithelial cells treated with vitamin D compounds. **(A)** Venn diagram comparing the number of proteins identified by mass spectrometry in lysates of MAC-T cells treated for 24 h with isopropanol (control) or with vitamin D_3_ (D, 50 nM), 25-hydroxyvitamin D_3_ (25D, 500 nM), or 1α,25-dihydroxyvitamin D_3_ (1,25D, 100 nM). Proteins exclusively identified in each treatment group are listed in **Supplementary Table 3**. **(B)** Volcano plots depicting differentially regulated proteins in pairs of conditions (i: D/Control; ii: 25D/Control; iii: 1,25D/Control; iv: 25D/D; v: 1,25D/D; vi: 1,25D/25D). The x-axis shows the log_2_ fold change and the y-axis the −log_10_ adjusted p-value. Proteins with a log_2_ fold change ≥ 1 (≥ 2-fold change) are shown as red dots (upregulated), whereas those with a log_2_ fold change ≤ −1 are shown as blue dots (downregulated). Statistical significance was defined at an adjusted p-value ≤ 0.05 (−log_10_(p) ≥ 1.3). **(C)** Heatmap showing the relative abundance of proteins with statistically significant differences (p ≤ 0.05) between treated cells (D, 25D, and 1,25D) and the control (isopropanol). Proteins (rows) and experimental conditions (columns) were hierarchically clustered using Euclidean distance and the complete linkage method based on z-score normalized abundance values. Four major protein clusters were identified and appear in yellow, green, orange, and gray.

A Venn diagram was generated to compare the number of proteins identified by MS in lysates of MAC-T cells treated for 24 h with isopropanol (control) or with D (50 nM), 25D (500 nM), or 1,25D (100 nM) (**Figure 4A**). A large shared proteomic core was found across all conditions, comprising 2,203 proteins. This indicates that the basal proteomic profile in MAC-T cells was highly conserved regardless of treatment. In addition, certain proteins were unique to each treatment, while others were shared by specific subsets of treatments. Cells treated with the vitamin D compounds exhibited both overlapping and treatment-exclusive proteins which were not present in the control. Put otherwise, the different compounds seem to have induced specific proteomic changes while a central set of shared cellular proteins was preserved.

The Venn diagram revealed that a limited number of proteins was exclusive to each of the vitamin D treatments (**Figure 4A**). Detailed information is provided in **Supplementary Table 3**. To facilitate interpretation, proteins in the table are classified according to the cellular components they belong to and the main biological processes and molecular functions in which they are involved. The focus is placed on pathways related to immunomodulation, host-pathogen interactions, and host-mediated mechanisms potentially associated with indirect antimicrobial activity.

Four proteins were exclusive to cells treated with D. Two of them are particularly relevant: a serine/threonine protein kinase (STK26, accession number A0AAA9SWA1) and the CCR4-NOT transcription complex subunit 3 (E1BCS1). The former is implicated in the regulation of apoptosis, oxidative stress responses, and intracellular signaling pathways. The latter is related with post-transcriptional control of gene expression, including mRNA stability and degradation.

On the other hand, out of the two proteins solely identified in cells treated with 25D, adaptor protein complex 2 subunit mu 1 (AP2M1, A0A452DIL3) is especially important here because of its central role in clathrin-mediated endocytosis and receptor trafficking.

Finally, a single protein was unique to treatment with 1,25D. This is the 14-3–3 beta/alpha protein (YWHAB, P68250), known to participate in intracellular signal transduction, regulation of the cell cycle and apoptosis, and metabolic control.

The data for proteins shared by all cells (treated and control) were subjected to pairwise comparisons, which were then used to build volcano plots (**Figure 4B**). The aim was to identify those proteins whose abundance was significantly different after 24 h of exposure to D or the two metabolites. **Table 4** shows the complete list of differentially regulated proteins identified in each comparison, together with their associated fold changes and adjusted *p*-values. There was an overall predominance of upregulated proteins over downregulated ones across all conditions. As indicated by the fold changes, only a small subset of differentially expressed proteins was markedly modulated, whereas most underwent relatively modest modifications. Those which were significantly regulated were classified according to gene ontology, molecular functions, biological processes, and biological pathways (**Supplementary Table 4**).

**Table 4:** Differentially regulated proteins across all treatments according to pairwise comparisons.

Accession number	Description	−log(*p*-value)	Fold change
D/Control			
O02741	Ubiquitin-like protein ISG15	1.661	1.783
F1N009	2’-5’ oligoadenylate synthase	1.503	1.278
Q17QZ9	Interferon-induced protein with tetratricopeptide repeats 5	2.212	1.128
Q3SWX4	Glioblastoma amplified sequence	1.330	1.029
F1N7C1	HECT and RLD domain containing E3 ubiquitin protein ligase family member 6	1.591	1.578
A3KMX9	Cholesterol side-chain cleavage enzyme, mitochondrial	1.408	1.506
A0A3Q1N191	GCN1 activator of EIF2AK4	2.487	1.784
F6Q4D3	Ubiquitin like modifier activating enzyme 7	4,496	1.151
E1BDX8	Dynein cytoplasmic 1 heavy chain 1	2.303	1.729
A0A3Q1M986	Polypeptide N-acetylgalactosaminyltransferase	1.507	-1.033
F1MMM8	Large ribosomal subunit protein bL19m	2.426	-1.262
A0A3Q1MFE4	MICOS complex subunit MIC60	1.693	-1.602
25D/Control			
A2VE31	Sodium-coupled neutral amino acid symporter 2	2.175	1.223
A0A3Q1LK48	Cytochrome P450 3A	3.709	1.922
Q3SWX4	Glioblastoma amplified sequence	2.946	1.198
A0A3Q1LVL2	Heme oxygenase 1	1.931	1.005
A0AAA9T2S1	Cytochrome P450 family 24 subfamily A member 1 (CYP24A1)	2.281	3.806
A0A452DJ03	Von Willebrand factor A domain-containing protein 1	2.041	-1.079
A0A3Q1MFE4	MICOS complex subunit MIC60	2.761	-2.058
A0AAF6YVK9	Ras-related protein Rab-3	1.637	-1.134
A0AAA9S260	Kinesin light chain	2.808	-1.446
A0AAA9T5Q6	F-box protein 2	1.503	-1.200
1.25D/Control			
A0A3Q1LK48	Cytochrome P450 3A	3.960	2.085
F1MJ80	Nicotinamide phosphoribosyltransferase	1.493	1.261
A0A3Q1MNT5	Exocyst complex component 7	1.402	1.344
A3KMX9	Cholesterol side-chain cleavage enzyme, mitochondrial	1.825	1.415
G3X7J5	Torsin family 4 member A	1.330	1.072
A0A3Q1NLW9	VPS37B subunit of ESCRT-I	2.138	1.275
A0AAA9TP18	Small ribosomal subunit protein bS16m	2.713	1.483
A0AAF6Z8F0	Caspase-4 (CASP4)	2.649	1.925
A0AAA9T2S1	Cytochrome P450 family 24 subfamily A member 1 (CYP24A1)	2.385	3.517
A0A140T888	1-phosphatidylinositol 4-kinase	2.209	1.767
A0A3Q1M1E9	Glycerol-3-phosphate dehydrogenase [NAD(+)]	1.377	1.537
Q5E972	ORM1-like protein 2 (ORM1)	1.596	3.090
F1N053	C-terminal binding protein 2	2.234	-2.524
A0A3Q1MFE4	MICOS complex subunit MIC60	1.955	-1.959
25D/D			
A0AAA9T2G8	Adhesion G-protein coupled receptor G1	1.658	1.285
A0A3Q1LK48	Cytochrome P450 3A	2.596	1.553
A0AAA9RZM4	Anthrax toxin receptor	1.852	1.683
A0A3Q1MQS2	CXADR Ig-like cell adhesion molecule	1.564	1.114
Q08E42	Leucine rich repeat containing 8 VRAC subunit A (LRRC8A)	2.091	1.346
A0AAA9T2S1	Cytochrome P450 family 24 subfamily A member 1 (CYP24A1)	2.316	2.836
A0A3Q1NBH6	glutaminase	1.403	1.400
F1N647	Fatty acid synthase	1.683	-1.819
A0AAF6YKW6	Large ribosomal subunit protein uL3	2.015	-1.008
A0AAA9RW41	Talin 1	1.794	-1.575
A0A3Q1MCV5	Coatomer subunit alpha	1.593	-1.325
F1MX04	Eukaryotic translation initiation factor 4 gamma 1	1.864	-1.030
A0A3Q1MR38	type I protein arginine methyltransferase	1.406	-1.001
P35605	Coatomer subunit beta’	1.402	-1.034
H7BWW0	Coronin	1.697	-1.022
F6QJE8	Platelet-activating factor acetylhydrolase IB subunit alpha	1.760	-1.668
1.25D/D			
A0A3Q1LK48	Cytochrome P450 3A	2.801	1.717
F1MMM8	Large ribosomal subunit protein bL19m	2.151	1.246
A0AAA9RZM4	Anthrax toxin receptor	1.759	1.672
Q08E42	Leucine rich repeat containing 8 VRAC subunit A (LRRC8A)	1.785	1.142
A0A3Q1MMR9	Protein phosphatase 6 regulatory subunit 3 (PP6RS3)	1.683	2.758
E1BF95	Protein phosphatase, Mg2+/Mn2+ dependent 1F	1.830	1.079
F6PSX0	Interferon induced protein 35	2.414	1.163
A0AAA9T2S1	Cytochrome P450 family 24 subfamily A member 1 (CYP24A1)	2.659	2.547
A0A452DKP2	Enoyl reductase (ER) domain-containing protein	1.506	-1.188
Q3ZBZ1	45 kDa calcium-binding protein	1.731	-1.941
1.25D/25D			
A0AAA9S260	Kinesin light chain	3.242	1.436
A0AAF6DM13	Galactose-1-phosphate uridylyltransferase	2.364	-1.239
Q9XSC3	WD repeat-containing protein 44 (WDR44)	2.262	-2.777

Fold-changes and *p*-values are shown for each.

Mitochondrial protein MIC60 (A0A3Q1MFE4), a core subunit of the mitochondrial contact site and MICOS cristae organizing system, was consistently downregulated in all cells treated with D or either of the two metabolites (**Table 4**; **Figure 4B** i, ii, iii). This is an essential component of the inner mitochondrial membrane complex, responsible for maintaining crista junction architecture and for mediating contact sites between the inner and outer mitochondrial membranes.

Cytochrome P450-related proteins were upregulated in cells treated with 25D and 1,25D compared with control and D-treated cells (**Table 4**; **Figure 4B** ii, iii, iv, v). These are hemethiolate monooxygenases primarily localized to the endoplasmic reticulum membrane, with crucial involvement in vitamin D metabolism. Although most of them underwent fold changes not exceeding 1, high fold values (2.5-3.8) were recorded for cytochrome P450 family 24 subfamily A member 1 (CYP24A1, A0AAA9T2S1) (**Table 4**). This enzyme is localized to the inner mitochondrial membrane and interacts with both 25D and 1,25D via hydroxylation at C23 and C24. It initiates the catabolism of active vitamin D, generating progressively inactive metabolites and establishing a negative feedback loop, whereby increased levels of 1,25D enhance CYP24A1 activity and thus prevent excessive vitamin D signaling. In addition, CYP24A1 has been reported to have 25-hydroxylase activity toward vitamin D_3_, and is therefore critical for vitamin D activation.

Among those proteins which were differentially regulated by 1,25D, ORM1-like protein 2 (Q5E972) showed ~3-fold upregulation and caspase 4 (CASP4, A0AAF6Z8F0) ~2-fold upregulation *vs* control cells (**Table 4**; **Figure 4B** iii). Two other proteins were also higher in 1,25D-treated cells with respect to those exposed to D: phosphatase 6 regulatory subunit 3 (PPP6R3, A0A3Q1MMR9) (~2.8-fold higher) and interferon-induced protein 35 (IFI35, F6PSX0) (**Table 4**, **Figure 4B**).

Leucine rich repeat containing 8 volume-regulated anion channel (VRAC) subunit A (LRRC8A, Q08E42) was upregulated in both 1,25D- and 25D-treated cells with respect to treatment with D (**Table 4**; **Figure 4B** iv,v). Moreover, WD repeat-containing protein 44 (WDR44, Q9XSC3) was downregulated ~2.8-fold in 1,25D-treated cells when compared to those exposed to 25D (**Table 4**; **Figure 4B** vi).

A heatmap was generated based on the relative abundance of proteins showing statistically significant differences between the four experimental conditions. This made it possible to have a global overview of proteomic regulation patterns and to identify clusters of similarly abundant proteins across treatments.

Hierarchical clustering resulted in four main protein groups (**Figure 4C**). Overall, the responses induced by the hydroxylated metabolites were more similar to each other than to the one induced by D. The first cluster (yellow) comprises proteins whose abundance was reduced in response to the metabolites but remained relatively unchanged in control and D-treated cells. Conversely, the abundance of those in the second cluster (green) increased predominantly in 25D- and 1,25D-treated cells, but was lower in the control and after exposure to D. Together, these clusters suggest that the two vitamin D metabolites had similar coordinated effects on protein regulation, and that these effects were more pronounced than those of D. The third cluster (orange) is made up of proteins whose abundance was higher in the control and lower in all the vitamin D-related treatments, which suggests this might be a general effect of such treatments. The proteins in the last cluster (gray) were more abundant in cells treated with the hydroxylated metabolites and less so in those exposed to D.

Proteins in the green cluster are found in the plasma membrane, cytoplasm, nucleus, mitochondria, and endomembranous system (**Supplementary Table 5**). Functional enrichment showed an overrepresentation of vitamin D metabolism/catabolism pathways (i.e. A0A3Q1LK48, A0AA9T2S1), epithelial homeostasis (translation, cell cycle, apoptosis, mitochondria) (i.e. A0AA9TGS6, Q2KIT4, F1N3F2, A0A3Q1LZ02, A4IFN6), innate immune signaling (interferon-responsive, TLR/MyD88, antiviral) (F1N3F2), and endocytic/autophagy processes (clathrin endocytosis, endosomal transport, autophagosome assembly) (i.e. A4IFN6, A0A3Q1NNG9) (**Supplementary Table 5**).

In summary, the proteomic analysis identified a conserved core of 2,203 proteins across conditions (**Figure 4A**). Compound-specific exclusives included STK26 (D), AP2M1 (25D), and YWHAB (1,25D) (**Supplementary Table 3**). The volcano plots revealed between three and 16 significantly regulated proteins per pairwise comparison (**Figure 4B**, **Table 4**). Among those that were upregulated, there were CYP24A1 (2.5-3.8-fold by 25D/1,25D), ORM1 (~3-fold by 1,25D), CASP4 (~2-fold by 1,25D and in the control), PPP6R3 (~2.8-fold by 1,25D/D), IFI35 (by 1,25D/D), and LRRC8A (by 25D/1,25D with respect to D). On the other hand, MIC60 was consistently downregulated in all vitamin D-related treatments with respect to the control, and WDR44 was downregulated ~2.8-fold by 1,25D with respect to treatment with 25D. Heatmap clustering confirmed a shared response elicited by the two hydroxylated metabolites, which consisted mainly in enhancing the abundance of proteins involved in vitamin D catabolism, epithelial homeostasis, innate immune signaling, and vesicular trafficking (**Figure 4C**; **Supplementary Table 5**).

### 3.5 25D reduces *S. aureus* internalization into MAC-T cells

**Figure 5:**
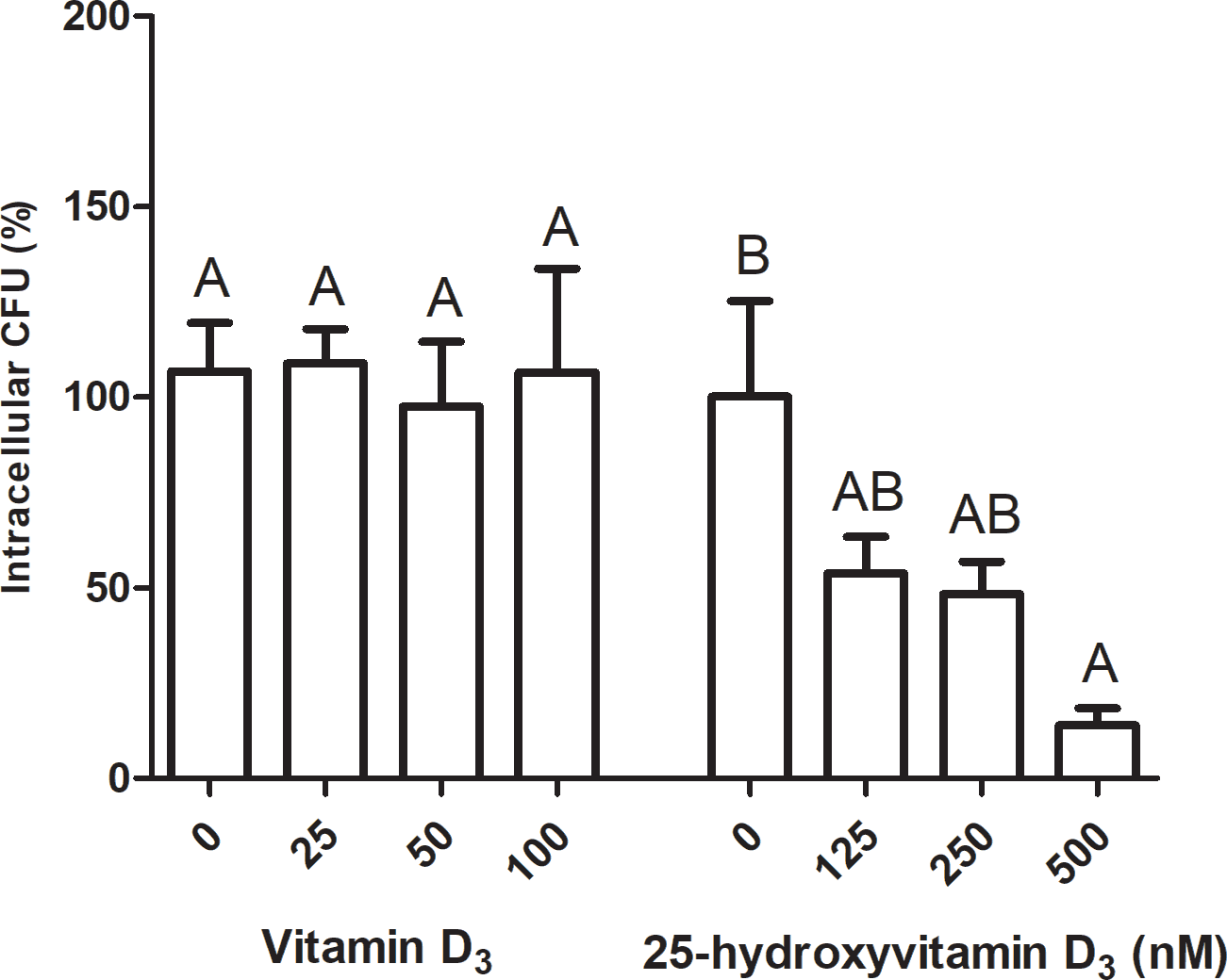
Effects of vitamin D and 25-hydroxyvitamin D_3_ on *S. aureus* internalization into bovine mammary epithelial cells. MAC-T cells were pretreated for 24 h with vitamin D_3_ (25, 50, or 100 nM) or 25-hydroxyvitamin D_3_ (125, 250, or 500 nM) and subsequently infected with *S. aureus* V329. Intracellular bacteria were quantified with a gentamicin protection assay. Internalization was expressed as the percentage of recovered intracellular colony-forming units (CFU)/mL relative to vehicle-treated control cells, which were considered to represent 100% internalization. Data are presented as mean ± SE. One-way ANOVA was performed, followed by Bonferroni’s *post hoc* test. Mean values were considered significantly different at p < 0.05. Different letters indicate statistically significant differences within each treatment group.

Pretreatment of MAC-T cells with D at 25, 50, or 100 nM for 24 h did not significantly affect the internalization of *S. aureus* V329, as shown by the similar numbers of intracellular colony forming units (CFU)/mL recovered from treated and control cells (**Figure 5**). Pretreatment with 125 nM or 250 nM of 25D did not produce significant differences in this parameter either. However, at 500 nM there was a statistically significant decrease in the number of intracellular CFU compared with the control (**Figure 5**).

## 4 Discussion

Vitamin D and its metabolites are known to have immunomodulatory properties, but their antimicrobial and antibiofilm activity remains underexplored. In the present study, no evidence was found of such activity for either D or 25D at the concentrations tested. Although slight strain-specific delays were registered in bacterial growth following treatment with 25D, inhibition was not measurable when compared with the untreated controls or with antibiotic treatment. Moreover, neither D nor 25D affected biofilm formation or reduced the biomass of pre-established biofilms. These findings are fully consistent with our previous results for 1,25D, which had no direct antimicrobial or antibiofilm effects on mastitis-associated *Staphylococcus* spp (Tiraboschi et al., 2024). Collectively, the data indicate that the three compounds exert minimal direct activity on these pathogens under planktonic or biofilm lifestyles *in vitro*. They also reinforce the notion that vitamin D and its metabolites may be useful mostly as modulators of the host immune response rather than as direct antibacterial agents.

Yue et al. (2017) reported that D and 25D inhibited *S. aureus* only at substantially higher concentrations than those tested in the present study (1000 ng/mL, equivalent to approximately 2500–2600 nM). This means that direct antimicrobial effects may require supraphysiological levels unlikely to be achieved *in vivo*. On the other hand, antibiofilm activity has been recently described for vitamin D against human Gram-negative clinical isolates, such as *Acinetobacter baumannii* and *Klebsiella pneumoniae* (Ganjo, 2024; Lutfi et al., 2024). However, there appears to be no other research into such effects against pathogens associated with bovine mastitis.

Both D and 25D were well tolerated by MAC-T cells in this study. Cell viability was not affected by D at any of the concentrations or points in time evaluated. A decrease in this parameter was recorded after 24 h of exposure to the highest concentration (2000 nM) of 25D, but it was mild and no longer detectable after 72 h. In short, no cytotoxic effects were observed for 25D at concentrations spanning the physiological range and higher, nor for D at supraphysiological concentrations commonly used *in vitro*.

Téllez-Pérez et al. (2012) likewise observed that MAC-T cell viability was preserved upon exposure to D. In contrast, Yue et al. (2017) found that the growth of these cells was inhibited after 24 h of treatment with approximately 5200 nM (2000 ng/mL) of the same compound. They also observed that concentrations above ~25 nM (10 ng/mL) of 25D decreased cell viability, with markedly stronger cytotoxicity at concentrations ≥ 2500 nM (≥ 1000 ng/mL) after 6 days. The present study tested lower concentrations of D (0–200 nM) and 25D (0–2000 nM) for shorter durations (24–72 h). As described above, no cytotoxic effects were detected, which highlights the concentration- and time-dependent nature of vitamin D-mediated effects on cell viability. These *in vitro* findings agree with the results of earlier *in vivo* tests by our group, in which intramammary administration of D (100 μg), 25D (250 μg), and 1,25D (10 or 30 μg) was well tolerated and elicited no adverse effects in Holstein cows (Tiraboschi et al., data not shown). Similarly, Nelson and colleagues (2011, 2017, 2018, 2022) (Wells et al., 2022) reported the safety of intramammary administration of 25D (100 or 500 mg) and 1,25-dihydroxyvitamin D_3_ (10 mg) in the same breed. All of these data point to vitamin D_3_ and its metabolites being safe at biologically relevant doses, and encourage further investigation into their potential as immunomodulatory agents to manage bovine mammary health.

On the other hand, the two hydroxylated metabolites studied here elicited a selective transcriptional response in bovine mammary epithelial cells, which primarily involved enzymes participating in vitamin D metabolism. Treatment with 25D significantly increased the expression of 25-hydroxylase and, more prominently, that of 24-hydroxylase. For its part, 1,25D selectively upregulated the expression of 24-hydroxylase. Neither metabolite significantly affected 1α-hydroxylase or VDR transcript levels. The profile just described indicates that both treatments seem to induce the activation of epithelial vitamin D catabolic pathways, as part of a feedback mechanism that limits excessive vitamin D signaling rather than enhance local activation.

In our earlier experiments in MAC-T cells, treatment with 1,25D for 24 h induced 24-hydroxylase expression without significantly altering VDR levels. Once again, this supports the existence of a conserved epithelial regulatory circuit controlling vitamin D homeostasis in the bovine mammary gland (Tiraboschi et al., 2024). Yue et al. (2017) also reported increased 24-hydroxylase expression and unchanged 1α-hydroxylase levels in MAC-T cells treated with 25D. They additionally described a significant downregulation of VDR. As mentioned before, our results are not fully consistent with this, but we did observe a decreasing trend for VDR after 12 h of treatment with 25D which did not reach statistical significance. The results by Téllez-Pérez et al. (2012) deviate more markedly from ours. In their study, treatment of bovine mammary epithelial cells with vitamin D was associated with an increase in the expression of both 25-hydroxylase and 1α-hydroxylase, and with a decrease in that of VDR. The divergence with our observations is likely explained by differences in the exposure time (12 h versus 24 h) and in the experimental models. Téllez-Pérez et al. used primary cells, which may retain greater metabolic plasticity and responsiveness to vitamin D. Instead, MAC-T cells appear to exhibit a more tightly regulated, epithelium-focused response centered on vitamin D catabolism.

The effects of vitamin D and its metabolites have been extensively explored at the transcriptional level in bovine mammary epithelial cells (bMEC/MAC-T). However, very little is known about such effects at the proteomic level from a comparative point of view. Proteomic analyses in cell lines and the bovine mammary gland have predominantly addressed physiological contexts or those subjected to stressors such as heat, lactation, or bacterial infection in the absence of vitamin D supplementation (Johnson et al., 2013; Zhu et al., 2023; Koch et al., 2024; Zhou et al., 2026). A few have looked into changes caused by 1,25D in porcine or human models (Cristobo et al., 2011; Wang et al., 2022). To the best of our knowledge, none have provided comparative insights into protein-level alterations effected by these compounds in bovine epithelial models that are directly relevant to mastitis and host-directed immunomodulatory strategies. This is a glaring gap considering the impact of the disease worldwide. In the present study, a large shared proteomic core was identified across all experimental conditions. Thus, exposure to the vitamin D compounds did not profoundly disrupt the basal proteomic landscape of MAC-T cells, but rather induced targeted and selective modulations. These observations support the concept of vitamin D as a fine regulator of epithelial homeostasis.

Although limited in number, the proteins which were exclusively detected under each vitamin D-related treatment are particularly informative from a functional perspective. Exposure to D induced proteins linked to post-transcriptional regulation (CCR4-NOT 3), apoptosis, oxidative stress, and intracellular signaling (STK26). In the case of the metabolites, 25D upregulated the endocytic machinery (AP2M1) and 1,25D enhanced YWHAB, which is associated with intracellular signaling, cell cycle regulation, metabolic control, and apoptosis. These specific signatures suggest that each compound engages partially divergent pathways despite signaling through the same nuclear receptor. Notably, AP2M1 was solely identified in 25D-treated cells. The AP2 unit is the best-characterized clathrin adaptor complex, and enables the clathrin-mediated entry of cargo from the plasma membrane into the endosomal system (Collins et al., 2002). Seeing that the invasion of *S. aureus* into bovine mammary epithelial cells is clathrin-dependent (Almeida et al., 1996; Latomanski and Newton, 2019), the presence of AP2M1 in cells exposed to 25D may contribute to explaining the reduction in bacterial internalization observed in our functional assays. This lends further strength to proteomic modulation being linked to host-pathogen interactions at the epithelial level.

The quantitative analysis of shared proteins revealed compound-specific signatures. MIC60 was consistently downregulated across all vitamin D treatments with respect to the control. Since intracellular pathogens manipulate MICOS subunits to promote mitochondrial fragmentation and ensure survival (Cheng et al., 2024), the downregulation of MIC60 may represent a host defense adaptation. Another important result was the marked upregulation of cytochrome P450 enzymes (led by CYP24A1) primarily in 25D- and 1,25D-treated cells, which mirrors the increased expression of 24-hydroxylase detected through RT-qPCR. This confirms that exposure to the metabolites resulted in the coordinated activation of vitamin D catabolic pathways, and validates the biological relevance of the proteomic findings.

Specific changes occurring in 1,25D-treated cells with respect to control cells included the ~3-fold upregulation of ORM1, a primary 1,25D–VDR response gene previously described in human monocytes and associated with the regulation of inflammatory deactivation and tissue homeostasis (Gemelli et al., 2013), and the ~2-fold upregulation of CASP4. CASP4 participates in bovine innate immune responses by mediating defense against bacteria, regulating inflammatory signaling pathways (including interleukin-18), and promoting proteolytic processing linked to programmed cell death mechanisms such as pyroptosis and apoptosis, thereby contributing to the control of infection and inflammatory balance. Moreover, PPP6R3 was ~2.8-fold higher in 1,25D- than in D-treated cells, which is indicative of NF-κB regulation (Heo et al., 2020). Taken together, these changes indicate that 1,25D reshapes epithelial innate immunity through a balanced modulation of pro- and anti-inflammatory pathways.

On the other hand, the downregulation of WDR44 in 1,25D-treated cells (~3-fold with respect to 25D) indicates metabolite-specific modulation of Rab11-associated vesicular trafficking. WDR44 is a Rab11 effector involved in regulating membrane recycling dynamics (Thibodeau et al., 2022). Therefore, its decreased abundance may alter endosomal trafficking pathways relevant to epithelial host–pathogen interactions. This finding supports the notion that 1,25D reshapes intracellular trafficking programs that could influence bacterial internalization (Tiraboschi et al., 2024) and downstream immune responses.

The coordinated response induced by the two hydroxylated metabolites is summarized by the green cluster on the heatmap. It stands in clear contrast with the one caused by D and encompasses proteins involved in vitamin D catabolism (CYP24A1/3A), epithelial homeostasis (cell cycle/apoptosis/mitochondria), innate immunity (IFN/TLR/MyD88), and vesicular trafficking (endocytosis/autophagy).

These proteomic findings build upon our earlier secretomic analysis of 1,25D-treated MAC-T cells and provide new insight into intracellular mechanisms underlying epithelial immune modulation (Tiraboschi et al., 2024). Whereas that study highlighted secreted proteins associated with antibiofilm activity against non-aureus staphylococci, including serpins, cystatin C, cathepsin B, and peroxiredoxin-1, the total cell lysate analysis presented here reveals complementary and compartment-specific intracellular patterns. Members of the serpin family (SERPINB1; A0A3Q1LN63) clustered with proteins that tended to decrease in cells treated with the hydroxylated metabolites relative to the controls. In contrast, in response to the same treatments cathepsin B (P07688) was upregulated and peroxiredoxin-like 2A (A0AAA9TRL1) varied minimally with respect to the controls. Together, these observations suggest that the metabolites differentially regulate the intracellular abundance and extracellular deployment of proteins related with host-defense mechanisms. They also underscore the importance of integrating secretomic and cellular proteomic techniques to fully capture the extent of epithelial antimicrobial programs.

The exploratory protein-protein interaction analysis based on the STRING database did not show highly interconnected networks among the differentially regulated proteins. This is consistent with the modest fold-changes observed, and suggests that the metabolites induced a distributed modulation of epithelial pathways rather than the activation of a single dominant signaling module. Functional enrichment assignments pointed mainly to processes related to vesicular trafficking, calcium and phosphate handling, apoptotic regulation, and cellular stress responses. Yet again, the changes support the concept that the metabolites were responsible for fine immunometabolic tuning in the mammary epithelial cells.

Earlier studies in bovine models reported the upregulation of several vitamin D-responsive molecules, including β-defensins, cathelicidins, and VDR. However, they were not detected in the present analysis. The absence of β-defensins is most likely attributable to the analytical scope of the untargeted shotgun proteomic approach, as these small cationic peptides may be better captured by dedicated peptidomics strategies. Cathelicidins exhibit very low basal expression in uninfected MAC-T cells and are typically induced at later stages (48–72 h) during staphylococcal infection, which rendered their detection unlikely during the 24-h treatment applied here (Kościuczuk et al., 2014). As for the non-detection of VDR, it is in keeping with the low basal protein abundance in non-specialized mammary epithelia and with the stable VDR mRNA levels recorded through RT-qPCR. This is further evidence in favor of a model in which vitamin D signaling is mediated primarily by pre-existing nuclear VDR pools rather than by *de novo* protein synthesis (Liesegang et al., 2008). On the other hand, the proteins that were identified in our analysis belong to expected functional categories, including enzymatic hydroxylases, immune signaling proteins, and regulators of epithelial homeostasis. In other words, despite the detection constraints mentioned before, the data confirm the biologically relevant engagement of vitamin D pathways (**Supplementary Table 6**). The validation of low-abundance effectors will require targeted strategies such as peptidomics, PRM/SRM, or immunoblotting in future studies.

Compound-specific effects were likewise evident in the *S. aureus* internalization assays. Though this parameter was not significantly affected by pretreatment with D, it was significantly reduced at the highest concentration of 25D. Therefore, the modulation of epithelial invasion appears to depend on what compound of vitamin D is used. Along the same lines, Yue et al. (2017) observed a reduction in the invasion of MAC-T cells by *S. aureus* following 24 h of pretreatment with 25D. In primary cells, however (Téllez-Pérez et al., 2012), it was pretreatment with D (0–200 nM) for 24 h that achieved a significant decrease in the internalization of the pathogen. The discrepancy between these results and ours may be ascribable to differences in the experimental models: primary bovine mammary epithelial cells and MAC-T cells are dissimilar in their differentiation status, receptor expression, and responsiveness to hormonal and immunomodulatory stimuli.

The present work sought to overcome the limitations of single-level approaches by combining functional assays, transcriptional analysis and proteomics with the aim of comparing the coordinated responses elicited by different vitamin D treatments in bovine mammary epithelial cells infected with mastitis-associated *S. aureus*. Compound-specific proteomic regulations of key vitamin D metabolic enzymes were thus revealed, backed up by the gene expression data. The treatments were additionally shown to modulate proteins involved in mitochondrial organization, endocytosis, and innate immune signaling. All these elements add up to novel mechanistic insights into how epithelial cells integrate vitamin D-dependent signals into their immune response. The proteomic shifts just mentioned should not be underestimated on account of their subtlety: non-professional immune cells like bovine mammary epithelial cells can profoundly alter host-pathogen dynamics through endocytosis (WDR44/AP2M1), mitochondrial regulation (MIC60), and immune signaling modulation. As the first attempt at comparing proteomic modulation by D, 25D, and 1,25D in MAC-T cells, this study positions vitamin D metabolites as complementary agents to combat antimicrobial resistance linked to mastitis management, in line with One Health principles.

The research does have limitations of its own, including the *in vitro* design, the use of a single epithelial cell line, and the absence of immune cell interactions that may amplify or shape vitamin D-mediated responses *in vivo*. Nevertheless, the findings remain biologically relevant for bovine mastitis, considering that mammary epithelial cells form the first line of defense against invading pathogens. Alongside the modulation of the proteome, the specific effects on bacterial internalization suggest that the tested compounds foster local cell-autonomous responses over systemic antimicrobial activity. Future studies should corroborate these observations in primary bMEC, co-cultures with immune cells, and *in vivo* models, as well as explore delivery strategies to optimize hydroxylated metabolite availability.

## Data Availability

The datasets presented in this study can be found in online repositories. The names of the repository/repositories and accession number(s) can be found in the article/**Supplementary Material**.
